# Metformin in gastrointestinal cancers and inflammatory bowel disease: Unraveling its mechanisms and therapeutic applications

**DOI:** 10.1016/j.isci.2026.114877

**Published:** 2026-02-02

**Authors:** Yanxi Li, Xingqi Guo, Xinxin Dong, Tong Xia, Siping Ma, Zhexian Liu

**Affiliations:** 1 Department of Colorectal Surgery, Cancer Hospital of China Medical University, Liaoning Cancer Hospital & Institute, Shenyang 110042, Liaoning, China

**Keywords:** therapeutics, human metabolism, cancer

## Abstract

Gastrointestinal diseases have become a global health concern with high incidence and prevalence. Considering that a great many patients with gastrointestinal cancers still suffer from tumor recurrence or distant metastasis even after surgical intervention or neoadjuvant chemotherapy, it is urgently needed to identify new therapeutic drugs. Metformin, originally derived from natural herbs, has been found to be beneficial for many diseases, including not only type 2 diabetes mellitus, cardiovascular disease, liver and kidney disease, and age-related diseases but also different types of cancers. Herein, we comprehensively review the roles and the underlying mechanisms of metformin in gastrointestinal cancers including colorectal, esophageal, and gastric cancers and also inflammatory bowel disease. By integrating the latest research findings, we systematically elaborate on how metformin exerts therapeutic effects on gastrointestinal diseases through the AMPK/mTOR pathway and the effects of combination therapies. This review may offer novel insights for developing therapeutic strategies against gastrointestinal malignancies and related diseases.

## Graphical abstract

**Figure:**
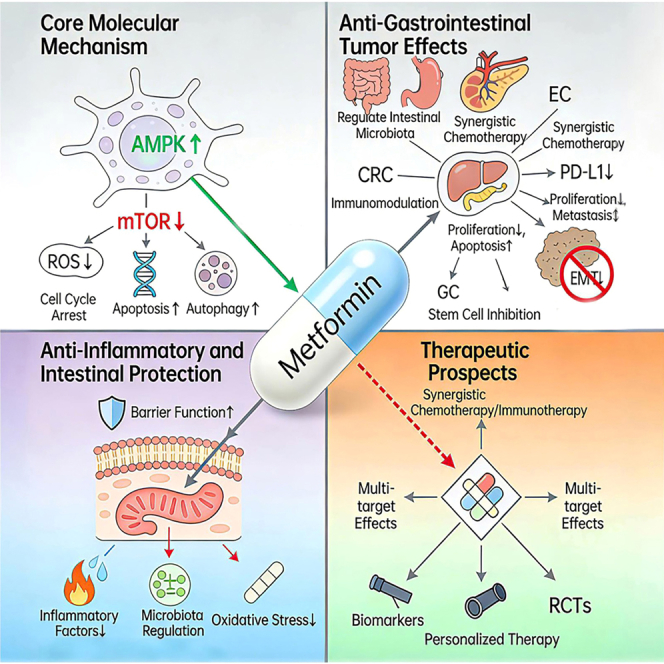

## Background

Gastrointestinal diseases pose a significant threat to global health and affect people of all ages, among which colorectal cancer (CRC) stands out as a particularly significant issue.^1^ CRC is a malignant tumor growing in the colon or rectum. Obesity and diabetes are one of the risk factors associated with CRC.^2^^,^^3^ The incidence of CRC has been elevated in recent years, which attributes to the changes of lifestyle, such as the increasing consumption of high-fat and low-fiber diets, as well as the lessened physical activities.^4^ Understanding the pathophysiology, risk factors, diagnosis, and therapeutic strategies of CRC as well as other gastrointestinal diseases is crucial for improving patient outcomes and reducing the global burden caused by these diseases.

**Figure 1:**
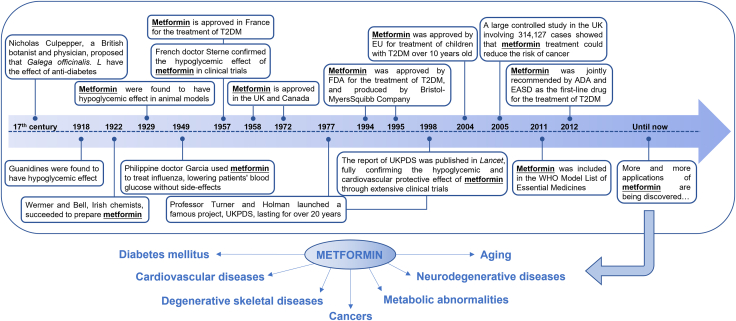
Drug history and clinical applications of metformin Metformin was originally derived from the plant galega (*Galega officinalis. L*) and was prepared in 1922 by Irish chemists. Since then, the effects of metformin have been fully studied in animal models and clinical trials. In 1994, metformin was approved by FDA for the treatment of T2DM. As the most widely used hypoglycemic drug, metformin has also been proved to show protective effects on other types of diseases, such as cardiovascular diseases, degenerative skeletal disease, neurodegenerative diseases, metabolic abnormalities, aging-related diseases, and cancers. ADA, American diabetes Association; EASD, European Association for the Study of Diabetes; EU, European Union; FDA, US Food and Drug Administration; UKPDS, United Kingdom Prospective Diabetes Study; WHO, World Health Organization.

Metformin, chemically designated as 1,1-dimethylbiguanide hydrochloride, is a widely used oral hypoglycemic drug and is originally derived from the plant galega (*Galega officinalis. L*) (Figure 1).^5^^,^^6^ The most extensively recognized application of metformin lies in the first-line clinical treatment of type 2 diabetes mellitus (T2DM).^7^ It operates by curtailing hepatic glucose production, augmenting insulin-mediated glucose uptake in the peripheral organs, and enhancing insulin sensitivity.^8^ The classic mechanism is to activate the AMP-activated protein kinase (AMPK) pathway, which inhibits hepatic gluconeogenesis and increases peripheral tissue uptake and utilization of glucose, thereby reducing blood glucose levels.^9^ Epidemiological studies have shown that the incidence rate of malignant tumors including gastrointestinal cancers in patients with T2DM is higher than that in the general population, such as gastrointestinal, liver, and breast cancers.^10^^,^^11^^,^^12^^,^^13^^,^^14^^,^^15^ Pharmacotherapy for T2DM, such as metformin, is also an influencing factor for the occurrence of malignant tumors. Metformin has been found to reduce the incidence rate and reflect better prognosis of malignant tumors.^16^^,^^17^^,^^18^^,^^19^^,^^20^

In recent years, the benefits of metformin for the treatment of various types of diseases have been reviewed and demonstrated, including hypertension,^21^ heart failure,^22^ cardiovascular disease,^23^ liver and kidney disease,^24^^,^^25^ age-related diseases,^26^ and cancers.^7^ In this manuscript, we comprehensively review the role and potential mechanisms of metformin in CRC and also demonstrate the updated roles of metformin in esophageal cancer (EC), gastric cancer (GC), and inflammatory bowel disease (IBD). In addition, we discuss the implications of these discoveries of metformin targeting the treatment of gastrointestinal cancers and the potential development of anti-tumor therapies.

## The role and mechanism of metformin in CRC

**Figure 2:**
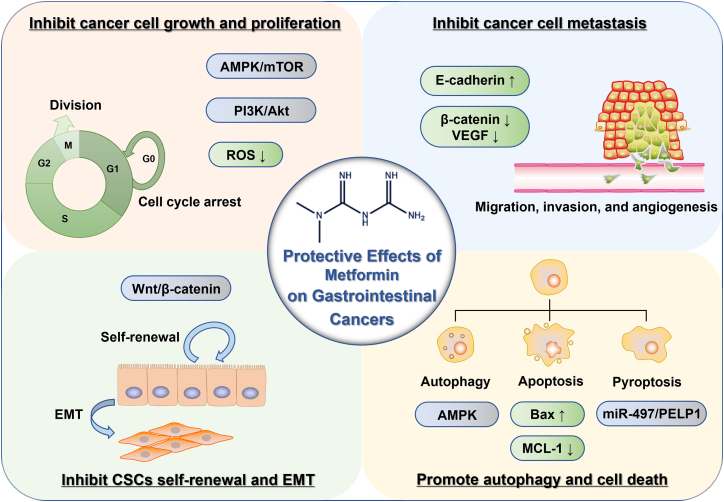
Protective effects of metformin on gastrointestinal cancers At present, many aspects of the antitumor effect of metformin on gastrointestinal cancers have been discovered, including CRC, EC, and GC. *In vitro* and *in vivo* studies have shown that metformin may exert antitumor effects on gastrointestinal cancers through various mechanisms. Metformin mainly exerts antitumor effects by inhibiting mitochondrial electron transport chain and ROS production, promoting the activation of AMPK, and inhibiting the mTOR signaling pathway. Metformin can induce cell-cycle arrest via the inhibition of PI3K/Akt signaling and thus impair the growth and proliferation of cancer cells. Metformin can also induce autophagy, apoptosis, and pyroptosis by activating AMPK or altering miRNA expression. In addition, metformin prevents the self-renewal of CSCs, impairs EMT, and inhibits the migration and invasion of cancer cells.

According to both *in vitro* and *in vivo* experiments, metformin has been demonstrated to impede the growth, proliferation, and metastasis of CRC cells, promote cell death, and prevent the epithelial-mesenchymal transition (EMT) process (Figure 2).

### *In vitro* and *in vivo* evidence of metformin in CRC

Substantial *in vitro* evidence has elucidated the therapeutic potential and molecular mechanisms of metformin in CRC models. The work by Zakikhani et al. found the dose- and time-dependent anti-proliferative effects of metformin on HT-29 cells through AMPK activation, a master regulator of cellular energy homeostasis.^27^ Another study demonstrated the transient growth suppression of metformin in multiple CRC cell lines (HT29, HCT116, and HCT116 p53^−/−^), mediated through a tripartite mechanism involving AMPK activation, oxidative stress, and consequent mTOR pathway inhibition.^28^^,^^29^ Notably, metformin was found to target mitochondrial complex I activity, resulting in mitochondrial membrane potential dissipation and subsequent reactive oxygen species (ROS) generation.^28^ These interconnected mitochondrial perturbations, comprising bioenergetic disruption and oxidative stress, collectively mediate the anti-proliferative effects of metformin in CRC. Metformin could block the activation of transforming growth factor β (TGF-β) signaling by targeting INHBA, thereby downregulating the activity of the PI3K/Akt pathway, thus leading to a decrease of cyclin D1 and cell-cycle arrest to inhibit the growth of CRC.^30^ A study found that metformin preferentially accumulates in KRAS-mutant CRC cells in the culture of primary cells and patient-derived xenografts (PDXs). Mechanistically, the mutated KRAS oncogene is highly methylated and silences the expression of multidrug and toxic compound efflux 1 (MATE1), which is a specific channel that excretes metformin from cancer cells by upregulating DNA methyltransferase 1 (DNMT1).^31^ Metformin effectively sensitized DLD-1, HT29, Colo205, and HCT116 human CRC cells to the pro-apoptotic activity of tumor necrosis factor-related apoptosis-inducing ligand (TRAIL).^32^ Metformin also upregulated Bax and downregulated anti-apoptotic myeloid cell leukemia 1 (MCL-1) levels in CRC cells, contributing to TRAIL-mediated cell death.^32^

In addition, *in vitro* evidence showed that the combination of different approaches targeting mTOR kinase or AMPK may be an effective strategy for treating CRC.^33^^,^^34^^,^^35^^,^^36^^,^^37^ For instance, 14-3-3zeta-overexpressing HCT15 and SW480 CRC cells were more sensitive to metformin, which might enhance the anti-tumor effect of metformin in CRC.^34^ Metformin enhanced 5-aminosalicylic acid (5-ASA)-induced CRC cell death by significantly increasing the activation of oxidative stress and apoptosis,^36^ implying the synergistic effect of metformin and 5-ASA combination on the treatment of CRC. MicroRNAs (miRNAs) can also improve the sensitivity of CRC cells to the anti-tumor effect of metformin.^38^^,^^39^ The combination therapy of metformin and TRAIL could not only lower the expression of MCL-1, *p*-JAK2, and p-STAT3 but also synergistically inhibit the proliferation of human CRC cells and promote cell apoptosis.^32^

Apart from *in vitro* evidence, it has been observed in mouse and rat models that metformin dose dependently inhibited the growth of colon tumors,^40^^,^^41^^,^^42^^,^^43^^,^^44^^,^^45^^,^^46^^,^^47^ suggesting that metformin is also effective for colorectal carcinogenesis *in vivo*. It was firstly demonstrated that metformin was able to alleviate the effect of high-energy diet on accelerating tumor growth in MC38 xenograft mice and could reduce insulin levels, AKT phosphorylation, and FASN expression.^40^ Metformin could delay tumor onset in mice with *p53* mutations and curb the development of diet-induced CRC in other animal models, which inhibited the proliferation of colonic epithelium by suppressing the mTOR signaling pathway.^48^ A study using mouse MC38 CRC model revealed the role of metformin in modifying tumor microenvironment metabolism, which could enhance the response to PD-1 blockade immunotherapy.^49^ Broadfield et al. demonstrated that in mice on a high-fat diet, metformin reduced tumor growth by altering the gut microbiome.^50^ It was found that transferring the gut microbiome from the metformin-treated mice to drug-free, high-fat mice suppressed the growth of murine CRC tumors. Increased levels of short-chain fatty acids, such as butyrate and propionate, were detected in the tumors, which might be linked to the downregulation of highly activated T cell clusters.^51^ A preclinical study found that metformin suppressed liver metastasis in a CRC xenograft mouse model by activating AMPK to inhibit mTOR phosphorylation.^52^ Moreover, the antitumor effect of metformin may be dependent on immune systems. Huang et al. revealed that metformin could stimulate CD8^+^ T cell function in CRC mouse model by reprogramming tryptophan metabolism,^53^ suggesting that metformin may be a potential immunotherapy strategy for CRC.

Combination therapy is a common treatment approach for CRC (Table 1). Chemical drugs including metformin combined with commonly used clinical drugs, herbal monomers, gene therapy, checkpoint blockade antibodies, and protein inhibitors can restore tumor sensitivity to chemotherapy drugs.^54^ Metformin is able to enhance anti-tumor immunity by regulating gut microbiota and can be used in combination with anti-PD-L1 antibodies to enhance anti-tumor effects.^55^ *In vivo* studies suggest that metformin combined with adjuvant chemotherapy may lead to a better prognosis. In colon cancer rat model induced by 1,2-dimethylhydrazine (DMH) and colitis-related colon cancer mouse model induced by DMH-dextran sulfate sodium (DSS), the combination of vitamin D3 and metformin exhibited a synergistic effect on the development of early colon tumors.^56^ In a DMH-induced CRC model, metformin plus oxaliplatin reduced DMH-induced CRC in both diabetic and non-diabetic mice by impairing tumor angiogenesis.^47^ Besides, the combination of metformin, 5-fluorouracil (5-FU), and oxaliplatin was highly effective in inhibiting the growth of recurring colon tumors in SCID mouse xenograft models of chemo-resistant HCT-116 and HT-29 cells.^57^ Likewise, metformin exhibited chemosensitization potential in PDX models when combined with 5-FU or 5-FU plus oxaliplatin regimens. The combination therapy significantly attenuated tumor progression through AMPK-driven molecular pathway or Hedgehog signaling pathway, concomitant with marked reduction of cancer stem cell (CSC) subpopulations of cancer cells.^57^^,^^58^^,^^59^ Combined with temsirolimus, metformin inhibited tumor growth and suppresses proteins related to mTOR, apoptosis, and EMT.^60^ When combined with 5-FU, metformin also inhibited the metastasis and proliferation of CRC.^61^ Treating CRC xenograft mice with metformin alone could reduce tumor proliferation and metastasis.^61^ Besides, the use of statin and metformin can provide a synergistic effect on gastrointestinal malignancy outcomes.^62^

**Table 1:** Combination approaches of metformin in cancers reviewed in this paper

Reference Num.	Year	Animal model	Combination approach	Effect
Saito et al.^19^	2016	CRC	metformin and TRAIL	inhibit the proliferation of human CRC cells and promote cell apoptosis
Tseng^21^	2023	CRC	metformin and mTOR inhibitor PP242	inhibit CRC cells *in vitro*
Bell et al.^24^	2016	CRC	metformin and 5-ASA	promote cancer cell death
Ding et al.^34^	2014	DMH-induced CRC mouse model	metformin and oxaliplatin	impair tumor angiogenesis in both diabetic and non-diabetic mice
Bekusova^42^	2015	DMH-induced CRC rat model and DSS-induced CRC mouse model	metformin and vitamin D3	inhibit the development of early colon tumors
Hosono et al.^43^	2014	SCID mouse xenograft models of chemo-resistant HCT-116 and HT-29 cells	metformin, 5-FU, and oxaliplatin	inhibit the growth of recurring colon tumors
Jia et al.^44^	2017	PDX models of CRC	metformin, 5-FU, and oxaliplatin	attenuate tumor progression
Khalili-Hezarjaribi et al.^45^	2017	solid tumors and lymphomas	metformin and temsirolimus	inhibit tumor growth
Koh et al.^46^	2020	CRC	metformin and 5-FU	inhibit the metastasis and proliferation of CRC
Jung et al.^63^	2025	EC	metformin combined with surgical operation and neoadjuvant radiotherapy	improve the overall survival of patients

Collectively, numerous *in vitro* and *in vivo* studies have demonstrated the tumor-suppressive effects of metformin in CRC. However, the dose of metformin commonly used in the preclinical study is far higher than the serum level (0.6 ± 0.5 mg/L) observed in diabetes, making it unclear whether these preclinical results are suitable in clinical treatment. The ongoing and future research studies should provide more evidence of the benefits of metformin combination therapy for cancer.

### Clinical evidence of metformin in CRC

As noted before, metformin, the first-line treatment for T2DM, is emerging as a potential chemopreventive agent for cancer. T2DM has been observed to be associated with an increased risk of developing CRC,^64^ whose incidence rate is about 1.2 times that of non-diabetes patients.^65^ Observational and preclinical studies have shown that metformin possesses anti-tumor effects on solid tumors including CRC. Epidemiological studies have shown that patients with diabetes not only have a lower risk of developing tumors on metformin treatment but also, when diagnosed with CRC, have a reduced mortality risk.^66^^,^^67^ Numerous clinical research findings have indicated that the cumulative duration and cumulative dose of metformin treatment are associated with a reduced incidence rate of cancer in diabetic populations, which also leads to more favorable outcomes in cancer patients. Specifically, long-term metformin use was linked to a decreased CRC risk in diabetic patients.^68^ Likewise, a cohort study involving 47,597 patients with T2DM found that metformin significantly reduces the risk of CRC in patients with T2DM in a dose-dependent manner among the Taiwanese population.^69^

Emerging epidemiological and clinical data have begun to delineate the potential chemopreventive role of metformin across CRC stages. Rigorous clinical studies demonstrate that metformin administration correlates with attenuated risks of CRC tumorigenesis, progression, and metastatic dissemination, suggesting the protective mechanisms. It was firstly reported that metformin was able to safely and directly suppress both colorectal epithelial proliferation and the formation of aberrant crypt foci at a dosage of 250 mg/day for 1 month, thus suggesting the inhibitory effect of metformin on CRC development.^70^ Numerous meta-analyses combining case-control and cohort studies indicated that metformin was related to a low risk of developing CRC among patients with or without diabetes.^71^^,^^72^^,^^73^^,^^74^^,^^75^^,^^76^^,^^77^^,^^78^ For instance, a case-control study by Sehdev et al. found that in the US, diabetic patients using metformin for 12 months had a 12% reduced risk of developing CRC.^77^ In studies in Italy and Spain, CRC prevalence was linked to diabetes, and metformin could reduce CRC risk (odds ratio [OR], 0.47; 95% confidence interval [CI], 0.24–0.92), while insulin increased it (OR, 2.20; 95% CI, 1.12–4.33).^76^ Cardel et al. summarized from 13 meta-analyses and 12 observational and 1 randomized study that metformin reduced CRC risk by 17% (OR, 0.83; 95% CI, 0.74–0.92).^79^ Some studies have also investigated the effects of metformin on non-diabetic CRC patients.^63^ A phase 2 clinical study with 50 refractory metastatic CRC patients enrolled demonstrated that 11 of 50 patients showed tumor stabilization after an 8-week administration of metformin combined with 5-FU.^80^

### Controversies and challenges

Although various clinical studies have observed the positive effect of metformin on protecting against CRC, there are still some contradictory observations in some studies. It was found that the administration of metformin in combination with adjuvant chemotherapy in patients with stage III CRC with diabetes resulted in similar overall survival and recurrence time compared to those without administration of metformin.^81^ Besides, a case-control analysis of 920 diabetes patients in the UK showed that high intake of metformin significantly increased the risk of CRC in men.^82^ Even though the random-effects model is accountable for a segment of the inter-study heterogeneity, the divergence of numerous additional confounding variables in the study design has likewise been imputed to the inter-study controversies, such as the provenance of the study population, the methodologies for determining diabetes and metformin exposure, as well as the dose and treatment duration of metformin. Statistically significant dose-response association between the use of metformin and a reduced risk of CRC was observed in different research studies.^79^^,^^83^ Metformin administration sequencing together with chemotherapy is also an important aspect to be considered, which would affect the efficacy of the treatment against CRC. Therefore, despite the incremental positive observations supporting the inhibitory clinical effect of metformin on colorectal carcinogenesis, larger sample sizes, more cohorts, more experiments of combined drugs, and dose optimization are still required in the upcoming preclinical and clinical trials.

## The role and mechanism of metformin in EC and GC

EC and GC are two common and highly malignant gastrointestinal tumors. The high incidence and mortality rates pose a serious threat to human health. Currently, the primary therapeutic approaches encompass surgical intervention, chemotherapy, radiotherapy, and targeted therapy. However, the clinical outcomes for patients with advanced-stage disease remain suboptimal. Metformin has been found to protect against these two types of gastrointestinal malignancies, mainly by modulating the proliferation, apoptosis, migration, and invasion of cancer cells (Figure 2).

### EC

It was observed that metformin suppressed the proliferation, promoted the apoptosis of KYSE450 EC cells without significant toxic effects, and also suppressed the growth of xenograft tumors in mice, mainly by inhibiting mTOR signaling both *in vitro* and *in vivo.*^84^ Subsequent *in vitro* experiments conducted in EC cell lines, including T.T, KYSE30, KYSE70, WHCO1, WHCO5, and SNO, demonstrated that metformin treatment significantly impaired cellular proliferation and triggered cell-cycle arrest.^85^^,^^86^ Metformin has been shown to suppress the proliferation of Eca-109 and TE-1 EC cells through the upregulation of USP7 expression.^87^ The inactivation of the STAT3-BCL-2 pathway promoted the crosstalk between apoptosis and autophagy, thereby leading to the inhibition of metformin-induced EC growth.^88^ In addition, numerous preclinical studies have extensively investigated the therapeutic potential of metformin in attenuating EC cell migration, invasion, and angiogenesis.^89^^,^^90^^,^^91^^,^^92^

Kobayashi et al. conducted comprehensive miRNA profiling in human EC cell models (T.T, KYSE30, and KYSE70).^86^ Pharmacological intervention with 10 mM metformin in KYSE30 cells revealed profound miRNA dysregulation, with high-throughput screening identifying 62 differentially expressed miRNAs (17 upregulated, 45 downregulated) following 72-h exposure. Unsupervised hierarchical clustering confirmed metformin-induced miRNA signature divergence, demonstrating compound-specific transcriptional reprogramming.^86^ Subsequent investigations employed system pharmacology approaches, integrating microarray datasets with Connectivity Map (CMAP) analyses to decipher miRNA-mediated regulatory networks. Notably, metformin was shown to activate tumor-suppressive pathways through the miR-375/TMCO3/PLA2G4A axis, revealing a novel regulatory mechanism in EC progression.^93^ Another study uncovered radiosensitization capacity of metformin via modulation of the miR-340-5p/KLF10 axis.^94^ Furthermore, Wang et al. identified apoptosis bypass mechanisms in refractory EC models. They demonstrated the ability of metformin to induce pyroptosis in advanced EC via the PELP1-dependent pathway, effectively circumventing conventional treatment resistance.^95^ In summary, metformin and agents that induce pyroptosis could potentially serve as alternative treatment options for EC that is resistant to chemotherapy and radiotherapy.

Accumulating evidence shows that metformin has the potential to modulate immune signaling pathways associated with inflammation in EC, for example, to downregulate the production of pro-inflammatory cytokines. Fan et al. conducted research using an N-nitroso-N-methylbenzylamine (NMBzA)-induced EC rat model and revealed that metformin could effectively reduce inflammation and the process of EC carcinogenesis. The underlying mechanism involved the suppression of inducible nitric oxide synthase (iNOS), cyclooxygenase-2 (COX-2), and interleukin (IL)-6, through the downregulation of the AMPK/mTOR pathway.^89^ Furthermore, Lu et al. investigated the impact of metformin on EC and discovered that it could inhibit the expression of programmed death-ligand 1 (PD-L1) through the IL-6/JAK2/STAT3 signaling pathway. It not only enhanced the function of T cells but also, in *in vivo* experiments, led to a decrease in PD-L1 levels. When metformin was combined with PD-1 inhibitors, it significantly enhanced the antitumor immune response.^96^ Overall, metformin exerts its influence on EC by suppressing PD-L1 expression via the IL-6/JAK2/STAT3 pathway, thereby effectively improving the antitumor immune response. These studies highlight the potential of metformin as a promising immunologic therapeutic agent in the context of EC treatment, opening up new avenues for further research and clinical applications.

Multiple meta-analyses suggested the anti-tumor effect of metformin on EC.^97^^,^^98^^,^^99^^,^^100^ For instance, metformin might inhibit the occurrence of EC and exhibit a positive impact on the overall survival of EC patients with diabetes. The combination of paclitaxel-based chemotherapy with metformin can also enhance the overall survival in cancer patients including EC.^101^ The administration of metformin combined with surgical operation and neoadjuvant radiotherapy may be more effective for improving the overall survival, compared to each therapeutic method alone.^99^ Contrary evidence exists, indicating that metformin may not significantly reduce the risk of EC in diabetic patients (95% CI, 0.60–1.28; *p* > 0.05).^102^ Nevertheless, geographical stratification revealed a distinct protective effect, with metformin demonstrating a statistically significant reduction in EC risk among Asian diabetic populations including Chinese and Korean populations (95% CI, 0.39–0.91; *p* = 0.02).^102^

### GC

Metformin reduced *in vitro* cell proliferation in MKN1, MKN45, and MKN74 GC cells by altering miRNA and inhibiting cell cycle-related molecules, which was further confirmed in BALB/c-nu/nu mouse model with subcutaneous xenografts of MKN74 cells.^103^ Metformin exerts multifaceted anti-tumor effects on GC through distinct molecular mechanisms. Synergistic effects between metformin and miR-365 have been demonstrated to induce GC cell apoptosis via the miR-365-pTEN-AMPK signaling axis.^104^ Mechanistically, metformin treatment in human AGS gastric adenocarcinoma cells could promote apoptosis through coordinated modulation of key signaling pathways, characterized by enhanced AMPK Thr172 phosphorylation and concurrent suppression of AKT Ser473, mTOR Ser2448, and p70S6K Ser424 phosphorylation.^105^ Furthermore, the AMPK/mTOR pathway-mediated inhibition of survival factors has been identified as a critical contributor to metformin-induced apoptosis in GC cells.^106^ The therapeutic potential of metformin is further enhanced when combined with chemotherapeutic agents, as evidenced by its synergistic effect with oxaliplatin in suppressing GC cell proliferation and inducing apoptosis through modulation of cell cycle regulators (cyclin D1) and apoptotic markers (Bcl-2, Bax, and caspase-3).^107^ Moreover, the combination therapy of metformin and doxorubicin has demonstrated robust effects in multiple types of cancer including GC, inhibiting tumor cell survival by inducing apoptosis or sensitizing to doxorubicin.^108^ Recent investigations have revealed that metformin counteracts the oncogenic effects of ADAMTS12, which is frequently upregulated in GC tissues and promotes tumor proliferation and glycolysis, with these findings being validated through comprehensive *in vitro* and *in vivo* studies.^109^ Besides, metformin demonstrated significant therapeutic potential by targeting GC stem cells, effectively impairing their self-renewal capacity and thereby inhibiting tumorigenesis in both experimental models.^110^

Metformin targets GC stem cells (CSCs), suppressing tumorigenicity and self-renewal. While initially shown to inhibit breast cancer CSCs,^111^ its effects extend to other types of cancers such as CRC.^110^ In GC, metformin reduces CSC proliferation in 2D/3D cultures and PDXs, downregulating CD44/Sox2 while upregulating differentiation markers, and delays tumor growth *in vivo.*^110^ Sekino et al. further demonstrated that metformin suppressed GC xenograft growth and NF-κB/Snail signaling linked to EMT.^112^ The Wnt/β-catenin pathway, frequently dysregulated in GC, drives EMT and CSC self-renewal. Metformin counteracts EMT in GC by suppressing β-catenin, vimentin, MMP9, and VEGF while upregulating E-cadherin, thereby reducing cell viability, migration, and invasion.^113^^,^^114^^,^^115^ Li et al. demonstrated metformin-induced downregulation of oncogenic long non-coding RNA (lncRNA) H19 in GC cells, activating AMPK, reducing MMP9, and inhibiting tumor invasion both *in vitro* and *in vivo*. Silencing H19 replicated the anti-tumor effects of metformin, highlighting its role in GC suppression.^114^ Additionally, Huang et al. identified an AMPK-LITAF-miRNA axis through which metformin inhibits the oncogene Bmi-1, suppressing EMT by downregulating metastasis-promoting miRNAs (hsa-miR-15a, -194, -128, -192).^116^ These findings collectively reveal the multi-targeted anti-CSC and anti-EMT mechanisms of metformin in GC.

The potential clinical application of metformin may be as a drug for reducing mortality rates and lowering recurrence rates. A large-scale Korean cohort study investigating diabetic GC patients who underwent gastrectomy revealed that prolonged metformin administration (>6 months) was significantly associated with reduced risks of both tumor recurrence and overall mortality.^117^ The impacts of metformin on reducing GC risk have been reviewed in recent years,^118^^,^^119^^,^^120^ which will not be covered in this manuscript.

## The role and mechanism of metformin in IBD

**Figure 3:**
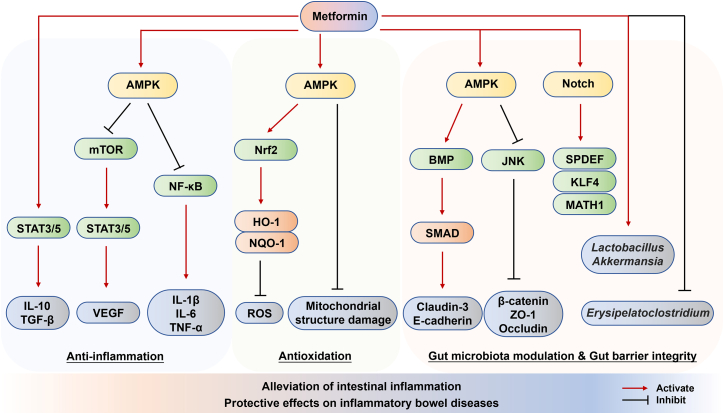
Protective effects and mechanisms of metformin in inflammatory bowel diseases Metformin is able to alleviate intestinal inflammation and thus protect against inflammatory bowel diseases. The anti-inflammation effect of metformin is mainly dependent on the upregulation of IL-10 and TGF-β, as well as the downregulation of pro-inflammatory cytokines in an AMPK-dependent way, such as IL-1β, IL-6, and TNF-α. Metformin also inhibits the production of ROS through the activation of AMPK/Nrf2. Metformin alters the gut microbiota composition by elevating the relative abundance of *Lactobacillus* and *Akkermansia* species while lowering *Erysipelatoclostridium* species, which alleviates the gastrointestinal inflammation and mucus barrier disruption. Moreover, metformin promotes the expression of tight junction proteins, such as claudin-3, E-cadherin, β-catenin, ZO-1, and occludin, thereby benefiting for gut barrier integrity.

Metformin also exhibits certain anti-inflammatory and protective effects in inflammatory gastrointestinal diseases such as IBD. Insulin resistance is commonly increased in IBD patients, while metformin alleviates insulin resistance and improves systemic insulin sensitivity.^121^^,^^122^ However, a lack of association between the effect of anti-diabetic drugs pioglitazone or rosiglitazone and the risk of IBD in Taiwanese patients with T2DM was observed according to two previous studies,^123^^,^^124^ suggesting that metformin may exhibit pleiotropic effects beyond pioglitazone and rosiglitazone. Extensive preclinical studies have demonstrated that metformin possesses dual anti-inflammatory and antioxidant properties while enhancing intestinal barrier function studied in both *in vitro* and *in vivo* models (Figure 3).

Prior research has demonstrated that in mucosal biopsies obtained from IBD patients, the levels of ROS were elevated, while the presence of antioxidants was diminished.^125^^,^^126^ In cellular models emulating IBD, metformin has been shown to effectively curtail the production of ROS, promote autophagy in IBD through AMPK signaling activation,^127^ and increase Nrf2 expression,^128^ which is a key regulatory factor of oxidative stress. Metformin upregulates antioxidant genes such as hemeoxygenase-1 (HO-1) and NAD(P)H/quinone acceptor oxidoreductase-1 (NQO-1).^128^ Oxidative stress can inflict damage on the mitochondrial structure and disrupt mitochondrial function and dynamics.^129^ Notably, metformin has the ability to mitigate hydrogen peroxide-induced mitochondrial fragmentation within a normal human colon cell line.^130^ This suggests that metformin may have a protective role in maintaining mitochondrial health, further contributing to its potential therapeutic benefits in the context of IBD.

The significant changes of the interaction between the gut microbiota and mucosal immune system are an important aspect of IBD. Metformin can alter gut microbiota to maintain the integrity of the intestinal barrier^131^ and can reduce the absorption of bile acids, thereby increasing the content of bile acids in the distal intestine and indirectly affecting the composition of the gut microbiota.^132^ Many studies have revealed that metformin can increase the production of short-chain fatty acids (SCFAs), such as butyrate and propionate in human,^131^^,^^133^^,^^134^^,^^135^^,^^136^ supporting that metformin can alter the composition of the gut microbiota by enriching SCFA-producing microbial communities and thereby mediating the anti-inflammatory function of metformin. Emerging evidence indicates that metformin administration can effectively modulate gut microbiota composition in murine colitis models, consequently attenuating intestinal inflammation and ameliorating IBD progression.^137^ In DSS-induced colitis mouse model, the biodiversity of gut microbiota lost during inflammation was restored under metformin treatment, which was also associated with a decrease in pathogenic *Escherichia shigella* and an increase in the abundance of *Lactobacillus* and *Akkermansia*.^138^ In diabetes and high-fat diet (HFD) rats, the enrichment of beneficial intestinal bacterial community was observed after long-term metformin treatment. The abundance of bacteria that produce SCFAs significantly increases, and the levels of lactic acid bacteria *Prevotella* and *Bacillus mucilaginosus* also increase, thereby improving the maintenance of metabolic processes and intestinal tissue homeostasis.^139^ A recent study developed metformin-loaded hydrogel microcapsules (MHMs) that allow for the controlled release of metformin to enhance its bioactivity. MHMs showed the ability to restore gut microbiome imbalance and normalize intestinal microbiome composition in IBD.^140^ Accumulating evidence from preclinical studies demonstrates that metformin exerts significant immunomodulatory effects in IBD models. Specifically, metformin administration has been shown to downregulate intercellular adhesion molecule-1 (ICAM-1) expression in experimental IBD models,^141^^,^^142^ while simultaneously upregulating anti-inflammatory mediators including IL-10 and TGF-β in colitis models.^143^ Furthermore, metformin treatment could significantly suppress the production of multiple pro-inflammatory cytokines, including but not limited to IL-1β, IL-6, IL-8, TNF-α, VEGF, IFN-γ, IL-13, IL-17, and IL-18, in various IBD experimental models.^137^

Intestinal epithelial barrier dysfunction is a key factor in IBD pathogenesis.^144^ Metformin reduces the epithelial permeability in inflamed tissue and colon cells.^145^ E-cadherin, an adherens junction protein vital for gut barrier integrity, has decreased expression in IBD patients’ colon tissues. Metformin mediated the upregulation and proper membrane localization of E-cadherin in inflamed colonic epithelium.^146^ The impact of metformin on cellular permeability and tight junction proteins are orchestrated through AMPK-dependent modulation and AMPK-induced JNK inhibition that strengthen the gut barrier.^141^^,^^145^ In line with *in vitro* findings, metformin could reduce gut epithelial permeability in IBD mouse models^145^^,^^146^ but showed diminished efficacy in mucosal healing stages.^147^ Metformin increased the expression of tight and adherens junction proteins such as E-cadherin but also decreased claudin-2, a pore-forming tight-junction protein, as observed in Crohn’s disease specimens and ileitis models.^146^^,^^148^ Metformin also enhanced mucus layer dynamics through goblet cell differentiation programs, evidenced by increased mucin2 expression and epithelial thickening in IBD-affected murine intestines through KLF4-mediated transcriptional reprogramming of gut epithelial progenitors.^149^

Epidemiological studies have yielded mixed results regarding the association between metformin use and IBD risk across different populations. A Taiwanese population-based study demonstrated a significantly lower incidence of IBD among diabetic patients receiving metformin compared to those treated with alternative anti-diabetic medications.^150^ On the contrary, a study from Denmark failed to establish a protective effect of metformin against IBD development in newly diagnosed diabetic patients initiating anti-diabetic therapy.^151^ A cohort study encompassing 1,323 ulcerative colitis (UC) patients and 1,278 Crohn’s disease patients provided compelling evidence that metformin use was significantly associated with improved clinical outcomes in established IBD cases, potentially mediated through its anti-inflammatory properties.^152^ These apparent discrepancies may be attributable to population heterogeneity, including variations in demographic characteristics (age and sex distribution), geographic factors, and disease phenotypes.

## Conclusions and future perspectives

In summary, metformin demonstrates considerable anti-tumor efficacy against gastrointestinal malignancies, with particularly pronounced effects in colorectal carcinogenesis. This therapeutic agent exerts its anti-tumor actions through multiple mechanisms, including the suppression of cellular proliferation, induction of apoptotic pathways, inhibition of metastatic processes (i.e., migration and invasion), and attenuation of angiogenic activity. These pharmacological effects are primarily mediated via activation of the AMPK pathway, precise modulation of the PI3K-Akt-mTOR signaling axis, and regulation of oxidative stress and inflammatory pathways. Evidence suggests that metformin is able to affect epigenetic processes through the regulation of AMPK activity. For instance, AMPK activates gene expression through phosphorylation and inactivation of HDAC and activation of HAT1, leading to an increase of histone acetylation.^153^ AMPK can also regulate gene expression through the ubiquitination of histone H2B.^154^ Metformin induces genome-wide alterations in the DNA methylation profile of cancer cells by positively regulating the activity of S-adenosyl-homocysteine (SAH) hydrolase,^155^ suggesting that metformin can act as a metabolic epigenetic regulator during the pathogenesis of gastrointestinal diseases. Despite promising anti-tumor and anti-inflammatory effects observed in various gastrointestinal tumors and inflammatory diseases, the precise mechanisms of metformin’s action require further elucidation. Future research directions should focus on several key areas: (1) investigating interindividual variability in metformin response and its mechanisms across different genetic backgrounds, (2) exploration of novel combination strategies with existing anti-cancer modalities, including targeted therapies and immunotherapies, (3) conducting large-scale, well-designed clinical trials to validate the long-term efficacy and safety profile of metformin in gastrointestinal malignancies and inflammatory conditions, (4) delving deeper into metformin’s roles in tumor microenvironment modulation, immune regulation, and epigenetic modifications, such as DNA methylation, histone modification, and chromatin remodeling, and (5) in-depth investigation of molecular mechanisms, particularly the intricate crosstalk between signaling pathways and tumor microenvironment dynamics.

To heighten the clinical relevance of the studies about metformin, it is essential to incorporate specific experimental designs. Multi-center randomized controlled trials (RCTs) are highly valuable in this regard, which allow for a more diverse patient population to be included in the research. Different centers may include patients with different genetic backgrounds, lifestyles, and co-morbidities. By gathering data from multiple centers, the results obtained are more generalizable and can provide more accurate insights into the effectiveness and safety of treatments in a broader clinical context. PDX model studies are also crucial, which involve transplanting human tumor tissues into immunodeficient mice. Through PDX model studies, researchers can test the efficacy of drugs in an *in vivo* environment that closely mimics human tumors, contributing to predicting how metformin and the relevant combination therapies will perform in actual human patients and thus bridging the gap between preclinical research and clinical application. Furthermore, biomarker development is an integral part of enhancing clinical relevance. For example, developing molecular signatures that can predict metformin efficacy is of great significance. Biomarkers can act as indicators to identify patients who are more likely to respond positively to metformin treatment. By analyzing a patient’s molecular profile, doctors can make more informed decisions about whether to prescribe metformin and what dosage to use. This personalized approach based on biomarker-guided treatment can improve treatment outcomes, reduce unnecessary drug exposure for non-responders, and optimize the use of medical resources. In conclusion, the inclusion of these specific experimental designs and biomarker development efforts is key to making research more clinically relevant.

In general, metformin, traditionally recognized as a hypoglycemic agent, exhibits considerable therapeutic potential in gastrointestinal oncology and inflammatory diseases. Sustained research endeavors will establish a more robust scientific foundation for its clinical application and may further expand its therapeutic utility in cancer management. Beyond the direct effects of metformin, attention should also be directed to the influence of drug dosage, combination therapy regimens, and patient lifestyle on treatment outcomes.

## Acknowledgments

This work was partially supported by the project of 10.13039/501100012131Department of Science & Technology of Liaoning Province (nos. 2023-MSLH-159 and 2024JH2/102600173) and the project of 10.13039/100007452Wu Jieping Medical Foundation (nos. 320.6750.2025-17-28, 320.6750.2024-17-1, and 320.6750.2025-13-101).

## Author contributions

Y.L., X.G., S.M., and Z.L.: methodology, investigation, and writing – original draft; X.D. and T.X.: methodology, investigation, and validation; S.M. and Z.L.: conceptualization, resources, writing – review and editing, supervision, and project administration.

## Declaration of interests

The authors declare no competing interests.
